# Translation, Cross-Cultural Adaptation, and Psychometric Validation of the Health Information Technology Usability Evaluation Scale in China: Instrument Validation Study

**DOI:** 10.2196/67948

**Published:** 2025-05-02

**Authors:** Rongrong Guo, Ziling Zheng, Fangyu Yang, Ying Wu

**Affiliations:** 1 School of Nursing Capital Medical University Beijing China; 2 Department of Hematology Peking University First Hospital Beijing China

**Keywords:** cross-cultural adaptation, digital health application, reliability, translation, usability, validity

## Abstract

**Background:**

The dramatic growth of digital health apps highlights an urgent need for rigorous usability evaluation tools. While the Health Information Technology Usability Evaluation Scale (Health-ITUES) has gained validation, a Chinese version has not yet been developed and validated.

**Objective:**

This study aimed to translate and culturally adapt the Health-ITUES into Chinese, customize it for both service consumers and professional health care providers, and evaluate its reliability and validity in the Chinese context.

**Methods:**

Following the Guidelines for the Process of Cross-Cultural Adaptation of Self-report measures, the Health-ITUES was meticulously translated and culturally adapted into Chinese version following 2 rounds of expert consultation. Subsequently, based on the SMART system, an intelligent and integrated older adult care model, the Chinese version of the Health-ITUES was customized into the care receiver version (Health-ITUES-R) and professional health care provider version (Health-ITUES-P). Older individuals and nurses participated in the validation testing conducted between December 2020 and February 2021, facilitated by the improvement of the COVID-19 pandemic and the timing preceding the Spring Festival, which ensured feasible recruitment and a sufficient sample size. In addition, the pandemic-driven increase in digital health app usage allowed us to assess usability in a relevant real-world health care setting. Content validity, internal consistency reliability, construct validity, convergent validity, discriminant validity, and criterion validity were used to evaluate the psychometric attributes of the Health-ITUES-R and Health-ITUES-P.

**Results:**

A Chinese version of the Health-ITUES comprising 20 items across 4 dimensions was formulated, informing the customization of the Health-ITUES-R and Health-ITUES-P. In total, 110 and 124 eligible older adults and nurses validated the customized Health-ITUES-R and Health-ITUES-P, respectively. Both versions exhibited satisfactory content validity (content validity index of items=0.83-1.00; content validity index of scale=0.99) and adequate internal consistency reliability (Cronbach α and McDonald ω>0.80 for the overall scale; >0.75 for individual items). Confirmatory factor analysis confirmed a 4D structure with acceptable construct validity, as indicated by model fit indices. Both the Health-ITUES-R and Health-ITUES-P showed satisfactory convergent validity (average variance extracted [AVE] value>0.5, composite reliability value>0.7), except for a slightly lower AVE value (0.478) for the second dimension of the Health-ITUES-R. Discriminant validity was supported, with the square root of AVE values exceeding correlation coefficients and the Hetereotrait-Monotrait ratio below 0.85. Furthermore, Pearson correlation coefficients for the perceived usefulness dimension, perceived ease of use dimension, and overall scale of the Health-ITUES-R and patient acceptance questionnaire for mobile health application were 0.587, 0.647, and 0.743 (all *P*<.01), demonstrating a significant correlation.

**Conclusions:**

The Chinese version of the Health-ITUES can be used as a valid and reliable tool to evaluate the usability of digital health apps for both care receivers and professional health care providers in the Chinese context.

## Introduction

The global realm of digital health apps has grown dramatically. This trend has been particularly notable since the onset of the COVID-19 pandemic in early 2020 [1,2]. As of the first quarter of 2021, more than 53,000 apps were available on the Google Play Store. This represents a notable increase of over 25% compared to about 42,000 apps on the same platform during the same period of the previous year [3]. These apps offer a feasible alternative to face-to-face communication between health care professionals and patients, assist health care professionals in diagnosing and managing various medical conditions by providing quick access to health data, disseminate health-promoting knowledge, and facilitate patients’ self-management, thus improving the efficiency of the health care system worldwide [4,5].

As digital health apps gain increasing popularity, the market is saturated with a diverse array of such apps, each varying in quality and functionality. Consequently, users frequently encounter difficulties in selecting the appropriate and truly useful apps that can enhance their health outcomes. A rigorous and standardized app rating system, implemented before the release of these apps to the major stores, empowers users to make well-informed decisions and fosters the healthy development of the app market [6]. However, the star rating and user reviews provided on the App Store are subjective and cannot accurately reflect the true usefulness and effectiveness of a digital health app [7]. In contrast, usability, defined as the extent to which users can use an app to achieve specific objectives with efficiency, satisfaction, and effectiveness in a specified usage context, is crucial to reflect the quality and efficacy of digital health apps [8,9]. Therefore, a rigorous and validated usability evaluation tool is urgently warranted to produce objective usability results for digital health apps before their release. This would enable consumers and researchers to promptly and efficiently select reliable apps.

Several questionnaires have been developed previously for usability testing, such as the System Usability Scale (SUS) [10], the Post-Study System Usability Questionnaire (PSSUQ) [11], the Software Usability Measurement Inventory [12], and the Computer System Usability Questionnaire (CSUQ) [13]. However, these questionnaires were originally designed with a focus on general information technology systems without considering the unique characteristics of digital health apps. These include the specialized content tailored to health needs, users’ high expectations regarding the accuracy of the information and functions, and the involvement of both service consumers (ie, patients who rely on the personalized chronic disease management apps to manage chronic diseases, access medical and health care information, and communicate with health care providers) and professional health care providers (ie, medical and nursing staffs involved in the personalized chronic disease management apps to monitor patients’ health status, provide medical advice, and coordinate treatment plans). Consequently, these usability evaluation tools prove challenging to reliably identify the specific problems that may arise when using digital health apps [14].

To bridge this gap, Yen et al [15] developed the Health Information Technology Usability Evaluation Scale (Health-ITUES) based on Bidshift, a web-based communication system for scheduling nursing staff to improve the efficiency and effectiveness of the staffing and scheduling process. Bidshift allows nurse managers to announce open shifts throughout the organization and staff nurses to request shifts. Consequently, the Health-ITUES was developed to assess nurses’ usability perceptions regarding the Bidshift system for shift requests at the task, individual, and organizational levels. It has been increasingly used as a validated tool to specifically evaluate the usability of digital health apps by clearly considering tasks [16]. The Health-ITUES also supports the customization at the item level to align with the specific tasks and expectations of the health systems while retaining comparability at the construct level [17]. The original English version of the Health-ITUES has been validated through exploratory factor analysis (EFA) and confirmatory factor analysis (CFA) among nurses [16] and community-dwelling adults with HIV [18]. Although a validated Korean version of Health-ITUES already exists [19], a Chinese version is currently unavailable. This absence creates a significant gap, especially considering the language, cultural norms, and the distinct regulatory framework and professional practices inherent in the Chinese health care system.

Therefore, this study aimed to translate and culturally adapt the Health-ITUES into Chinese, revise its customized parts to cater to both service consumers and professional health care providers, and evaluate its reliability and validity in the Chinese context.

## Methods

### Study Design

This study followed the guideline for the process of cross-cultural adaptation of self-report measures by Beaton et al [20]. The guideline offers a well-established and systematic framework to ensure rigor and validity of the cross-cultural adaptation of the Health-ITUES, minimizing potential biases and errors that might arise during the translation and adaptation processes. It also enables comparability with other studies that have followed the same or similar procedures and facilitates a more meaningful synthesis of research findings within the field. Before initiating the translation process, we obtained permission from the original author of the Health-ITUES via email to translate it into Chinese (refer to Figure S1 in Multimedia Appendix 1). This paper was reported in accordance with the Strengthening the Reporting of Observational Studies in Epidemiology (STROBE) checklist (Multimedia Appendix 2).

### The Original Health-ITUES

The Health-ITUES was originally designed to measure the usability of a web-based communication system for scheduling nursing staff. Derived from the health information technology usability evaluation model, a theoretical framework to guide usability evaluations of digital health technologies [21], the Health-ITUES has recently been increasingly used to accurately assess the usability of digital health apps [18,19]. The tool allows for customization of the items to match the specific tasks and expectations of the health systems. It comprises 20 items from 4D: quality of work life (3 items), perceived usefulness (9 items), perceived ease of use (5 items), and user control (3 items). Each item is rated from 1 (strongly disagree) to 5 (strongly agree) on a 5-point Likert scale [15]. The total scores range from 20 to 100, with higher scores indicating better perceived usability. The English version of the Health-ITUES demonstrated satisfactory reliability and validity, with Cronbach α coefficients and criterion validity indexes of 0.85-0.92 and 0.46-0.70, respectively [16].

### Translation and Cross-Cultural Adaptation of the Health-ITUES

#### Forward Translation and Synthesis of the Forward Translations

In total, 2 bilingual native Chinese speakers who were proficient in English and had passed the College English Test Band Six produced 2 forward Chinese translations of the Health-ITUES independently (T1 and T2). One of the translators was familiar with the Health-ITUES contents, while the other was unaware of the concepts being quantified. Subsequently, the 2 translations were meticulously reviewed for any ambiguity until a consensus was reached. In the process, we also invited a third translator to resolve disagreements. Through iterative comparison and refinement, a synthesized Chinese version, T3, was achieved.

#### Back Translation

The synthesized Chinese version T3 was independently back-translated into English versions (BT1 and BT2) by another 2 experienced translators who were native English speakers and had a good command of Chinese. Both of the back translators were blinded to the original Health-ITUES. The research team then compared the 2 back translations, analyzed the similarities and differences between them, and provided feedback to the original author of the Health-ITUES for verification. The basic information of the translators was summarized in Table S1 in Multimedia Appendix 1.

#### Cross-Cultural Adaption

All translated versions of the Health-ITUES (including the 2 forward translations, the synthesized version, and the 2 back translations) were thoroughly discussed and evaluated by a panel of 6 experts with varied research fields, encompassing older welfare technology, clinical nursing, nursing information, Chinese and American culture, and data science and engineering (refer to detailed information in Table S2 in Multimedia Appendix 1). The experts were invited to provide feedback on the accuracy of translation and professional terminology, readability, seamless integration with the language of the information system, comprehensibility for nonprofessionals, alignment with clinical practice, cultural appropriateness, and item retention. Through the adoption of a self-evaluation method (Table S3 in Multimedia Appendix 1), the authority coefficient (Cr) was determined by considering the familiarity coefficient (Cs) and the judgment coefficient (Ca). The formula for calculating the Cr is Cr = (Cs + Ca)/2 [22].

Based on their professional theoretical knowledge and practical experience, the experts evaluated each item independently on semantic equivalence, conceptual equivalence, experiential equivalence, and idiomatic equivalence. Any items with ambiguity were reworded until all expert queries were addressed, eventually resulting in the final Chinese version of the Health-ITUES.

### Validation of the Health-ITUES

#### Overview

We used the SMART system (known in Chinese as Aifuxing App), developed in the early stage, as a digital health App for the Health-ITUES validation. In short, the SMART system was primarily designed to facilitate personalized integrated home-based care for older people [23]. Considering that older individuals and professional care providers are the main users of the app, this study aimed to validate the effectiveness of the Chinese version of the Health-ITUES among both older people and professional health care providers.

#### Customization of the Care Receiver and Professional Health Care Provider Versions of the Health-ITUES

Based on the overall objectives and functional components of the SMART system, the research team engaged in multiple rounds of discussions to refine the customized components within the Chinese version of the Health-ITUES, formulating the initial care receiver (ie, Health Information Technology Usability Evaluation Scale—care receiver version [Health-ITUES-R]) and professional health care provider versions of the Health-ITUES (ie, Health Information Technology Usability Evaluation Scale—professional health care provider version [Health-ITUES-P]). Subsequently, a panel of 6 experts from pertinent fields (refer to specific details in Table S4 in Multimedia Appendix 1) were tasked with reviewing the 2 initial customized scales and assigning ratings for the correlation between each item and the corresponding dimension on a 4-level scale: 1=uncorrelated, 2=weakly correlated, 3=strongly correlated, and 4=highly correlated. The research team then iteratively modified the items according to expert suggestions until a consensus was reached among all experts on the finalized Health-ITUES-R and Health-ITUES-P.

#### Study Participants

The validation test was conducted in a geriatric ward of a comprehensive hospital in Beijing, China from December 2020 to February 2021. A total of 3 primary factors underlie our choice of this research time frame. First, during the COVID-19 pandemic, the hospital adopted a closed-door management strategy that strictly restricted inpatient numbers and prohibited nonhospital staff from entering. This situation persisted until December 2020, when the improving pandemic conditions enabled patient admissions and researchers’ entry for recruitment activities. Concurrently, a significant increase in patient arrivals guaranteed an adequate sample size. Second, the research period extended just before the Spring Festival, a major traditional Chinese festival, which further enhanced the robustness of the sample size to generate reliable results. In addition, the surge in digital health app use during the COVID-19 pandemic allowed us to assess usability in a relevant, real-world health care environment. Older adults were included consecutively if they (1) were aged 60 or older, (2) possessed normal communication and interaction abilities, (3) obtained at least a primary school education, (4) had an android-based smartphone for internet access, and (5) expressed willingness to participate. Older individuals with dementia or other mental illness were excluded to ensure comprehension of the scale items. To validate the Health-ITUES among professional health care providers, nurses, considered as the primary professional health care providers, were included. In-service nurses holding nurse qualification certificates were enrolled in the study if they had an android-based smartphone and were willing to participate.

### Ethical Considerations

The study followed the Declaration of Helsinki and received approval from the institutional review committee of the Capital Medical University (approval number 2015SY49U). Potential participants were thoroughly informed of the study’s objectives, methods, procedures, and the data that would be collected, as well as their right to discontinue their participation at any time without facing any adverse impacts. Only those who provided written informed consent were enrolled. In addition, to safeguard participants’ privacy, personal identifiers were stored in password-protected files, and only deidentified data were used for analysis and reporting purposes. In recognition of their contribution, each participant was rewarded with a small token valued at ￥20 (about US $3). The manuscript and supplementary materials were meticulously designed to exclude any information that could disclose the identities of individual participants.

### Instruments

#### General Information Collection

Demographic information including age, gender, education, and monthly income was collected from both older individuals and nurses. Besides, we gathered data on nurses’ professional titles and years of employment. To measure participants’ usage frequency of common functions on mobile phones, a mobile phone usage experience questionnaire was used. This questionnaire, derived from the questionnaire on computer experience, consists of 8 items, each rated on a 5-point Likert scale ranging from 1 (never) to 5 (very frequently). The total scores range from 8-40, with 8-16, 17-32, and 33-49 indicating low, moderate, and abundant mobile phone usage experience, respectively. The questionnaire exhibited satisfactory reliability, with a Cronbach α coefficient of 0.922 [24].

#### The Health-ITUES-R and Health-ITUES-P

The finalized Health-ITUES-C and Health-ITUES-P adapted from the Chinese version of the Health-ITUES were used to collect the perceived usability of the SMART system among older individuals and nurses, respectively. Respondents rate these items on a 5-point Likert scale from 1 (completely disagree) to 5 (completely agree). Total scores for both versions are calculated by summing the scores of each item, with higher scores reflecting better-perceived usability.

#### Patient Acceptance Questionnaire for the Mobile Health App

The patient acceptance questionnaire for the mobile health app was used as a reference standard to evaluate the criterion validity of the Health-ITUES. This questionnaire, designed to evaluate patients’ acceptance of mobile medical products, consists of 32 items covering 6D: usefulness, ease of use, trust, usage attitude, system interface, and usage tendency. Respondents provide ratings for each item on a 5-point Likert scale ranging from 1 (strongly disagree) to 5 (strongly agree), resulting in a total score of 160. The questionnaire had satisfactory reliability and validity, with the scale-content validity index (S-CVI), Cronbach α coefficient, and split-half reliability of 0.97, 0.96, and 0.99, respectively [25]. Given its focus on users of medical apps, the questionnaire was only administered to older people.

#### Data Collection Procedures

After providing a comprehensive explanation of the study’s purpose, significance, and procedures, the research team assisted the participants in downloading and installing the SMART system and completing registration and login. Training materials, including instructional videos and user manuals, were made available to the participants until they felt confident in using the app. Subsequently, the participants were required to use the SMART system independently for 24 hours to complete the assigned tasks before filling out the Health-ITUES-R, Health-ITUES-P, and patient acceptance questionnaire for the mobile health app as appropriate. During the 24-hour app-use period, the enrolled older participants and nurses were instructed to complete the assigned tasks independently, without seeking assistance or discussing related content with others. Continuous monitoring was conducted by the research team and nurses from the department who were not enrolled as participants. The detailed tasks assigned to older adults and nurses are listed in Table S5 in Multimedia Appendix 1.

#### Sample Size Calculation

To achieve adequate statistical power, the sample size should be 5-10 times the number of items [26]. With a total of 20 items in the Health-ITUES, the study necessitated a minimum of 100 participants. Taking a 20% dropout rate into consideration, at least 110 older individuals and nurses were needed for the study. The anticipated dropout rate of 20% was based on prior studies investigating app usability [27,28].

#### Statistical Analysis

Continuous data was tested for normal distribution by using the 1-sample Kolmogorov-Smirnov test and expressed as mean SD or medians with IQR as appropriate. For the between-group comparison, the student *t* test was used for continuous variables with normal distribution, while the Mann-Whitney *U* test was used for nonnormally distributed continuous data. Categorical variables were expressed as frequencies or proportions (%) and comparisons were conducted using chi-square or Fisher exact test as appropriate.

The content validity of the finalized Health-ITUES-R and Health-ITUES-P was assessed by the item-level content validity index (I-CVI) and S-CVI based on expert ratings. I-CVI is the ratio of the experts ranking the item for 3 or 4 scores, and the S-CVI is the average value of all the I-CVI scores [29]. A scale with an I-CVI of > 0.78 and a S-CVI of ≥ 0.90 is considered satisfactory [30].

The internal consistency reliability was determined by Cronbach α, McDonald ω, and corrected item-total correlation coefficient (CITC). Values of Cronbach α and McDonald’s ω ≥ 0.70 are considered adequate, while a value of CITC of <0.30 indicates a low correlation [31,32]. While the test-retest reliability could assess measurement consistency under consistent conditions, the fluctuating nature of users’ perceived usability of the digital health apps over time makes this indicator unsuitable [33]. Furthermore, due to inherent variations in user perceptions of the app’s usability, interrater reliability was not examined in the study.

CFA with maximum likelihood estimation was performed to explore the structure validity. The analysis provided standardized factor loading to estimate the relationship strength between items and dimensions [34], together with model fit indices, including χ^2^/df, root-mean-square error of approximation (RMSEA), root-mean-square residual (RMR), standardized root-mean-square residual (SRMR), parsimonious goodness-of-fit index (PGFI), parsimonious normed fit index (PNFI), and parsimonious comparative fit index (PCFI). Acceptable structure validity was evaluated using recommended cut-offs characterized as standardized factor loading of ≥0.60, χ^2^/df of <3, RMSEA of ≤0.10, RMR of ≤0.05, SRMR of ≤0.80, PGFI of ≥0.50, PNFI of ≥0.50, and PCFI of ≥0.50 [35].

To determine the convergent validity, the composite reliability (CR) and average variance extracted (AVE) were calculated through the Fornell and Larcker approach [36] with a CR ≥ 0.70 and AVE ≥ 0.50 indicating satisfactory convergent validity. The square root of the AVE exceeding each of its correlations with other dimensions indicates appropriate discriminant validity [37]. The discriminant validity was also tested by the Heterotrait-Monotrait ratio (HTMT), where a value <0.85 is acceptable [38]. In addition, the criterion validity between the Health-ITUES and patient acceptance questionnaire for mobile health apps was analyzed through Pearson correlation analysis, with correlation values of >0.50 deemed adequate [39].

Statistical analyses were performed using AMOS version 26.0 (IBM Corp) for CFA and SPSS version 26.0 (SPSS Inc) for the remaining analyses. A 2-sided *P* value of <.05 was considered statistically significant.

## Results

### Translation and Cross-Cultural Adaptation Versions of the Health-ITUES

The detailed summary of the forward translations T1 and T2, synthesized version T3, and back translations BT1 and BT2 was provided in Table S6 in Multimedia Appendix 1. Following the first and second rounds of consultations with the expert panel, a total of 6 and 5 modifications were made, respectively, to formulate the final Chinese version of the Health-ITUES. The dimension “quality of work life” was deemed inadequate in reflecting the corresponding items accurately. Under expert guidance, we changed it to “impact.” Further details of expert suggestions and specific modifications on the synthesized version T3 were summarized in Tables S7 and S8 in Multimedia Appendix 1. The Cr of the expert panel was 0.89, indicating a high level of expert authority.

### Validation of the Health-ITUES

#### Customization of the Health-ITUES-R and Health-ITUES-P

Based on the final Chinese version of the Health-ITUES, the research team proceeded to customize the Health-ITUES-R and Health-ITUES-P to align with the specific tasks and expectations of the SMART system after extensive discussions. Subsequently, according to the suggestions from the expert panel with a Cr of 0.95, the research team made revisions to 6 and 4 items in the initial Health-ITUES-R and Health-ITUES-Provider to formulate the finalized versions for further validation. The expert suggestions and revisions as well as the finalized Health-ITUES-R and Health-ITUES-P were shown in Tables S9-S12 in Multimedia Appendix 1.

#### Validation of the Health-ITUES-R and Health-ITUES-P

##### Baseline Characteristics of Older Individuals and Nurses

A total of 110 and 124 eligible older adults and nurses were included in the validation test, respectively. Table 1 showcases their baseline characteristics. The median age of the older participants was 67 (IQR 64-71) years with 67.27% (74/110) being male. The enrolled nurses were largely female (112/124, 90.32%) with a median age of 26 (IQR 24-28) years. Overall, 52.73% (58/110) of the older participants were reported to have limited experience in using mobile phones, while the rest (52/110, 47.27%) exhibited moderate experience. In contrast, nurses exhibited more experience in using mobile phones, with 91.13% (113/124) having moderate usage experience and 8.87% (11/124) possessing abundant experience. Furthermore, older people tended to spend less time on their mobile phones every day, with only 10% (11/110) using their mobile phones for more than 3 hours per day, while 69.35% (86/124) of nurses reported using mobile phones for more than 5 hours per day.

**Table 1:** Baseline characteristics of the enrolled older adults and nurses in the validation study.

Characteristics			Older adults (n=110)		Nurses (n=124)
Age (years), median (IQR)			67 (64-71)		26 (24-28)
Male, n (%)			74 (67.27)		12 (9.68)
**Education, n (%)**					
	Primary school and below	28 (25.45)		—^a^	
	Junior high school	51 (46.36)		—^a^	
	Senior high school and above	31 (28.18)		—^a^	
	Junior college or below	—^a^		63 (50.81)	
	Undergraduate or above	—^a^		61 (49.19)	
**Monthly income (￥)^b^, n (%)**					
	≤1000	16 (14.55)		—^a^	
	1001-3000	41 (37.27)		—^a^	
	3001-5000	42 (38.18)		—^a^	
	>5000	11 (10.00)		—^a^	
	≤5000	—^a^		29 (23.39)	
	5001-10,000	—^a^		66 (53.23)	
	>10,000	—^a^		29 (23.39)	
**Years of work experience, n (%)**					
	<4	—^a^		64 (51.61)	
	4-9	—^a^		50 (40.32)	
	>9	—^a^		10 (8.06)	
**Professional titles, n (%)**					
	Junior level	—^a^		93 (75.00)	
	Intermediate level	—^a^		29 (23.39)	
	Senior level	—^a^		2 (1.61)	
**History of chronic diseases, n (%)**					
	Hypertension	75 (68.18)		—^a^	
	Diabetes mellitus	37 (33.64)		—^a^	
	Hyperlipidemia	31 (28.18)		—^a^	
	Stroke	38 (34.55)		—^a^	
	Coronary heart diseases	9 (8.18)		—^a^	
**Usage experience of mobile phones, n (%)**					
	Less experience	58 (52.73)		0 (0)	
	Moderate experience	52 (47.27)		113 (91.13)	
	Abundant experience	0 (0.00)		11 (8.87)	
**Daily mobile phone usage duration, n (%)**					
	<1 hour	52 (47.27)		—^a^	
	1-3 hours	47 (42.73)		—^a^	
	>3 hours	11 (10.00)		—^a^	
	<5 hours	—^a^		38 (30.65)	
	5-7 hours	—^a^		64 (51.61)	
	>7 hours	—^a^		22 (17.74)	

^a^Not applicable.

^b^￥1=US $0.0071.

##### Reliability and Validity Evaluation Results

As shown in Table 2, the I-CVI for both the Health-ITUES-R and Health-ITUES-P ranged from 0.83 to 1.00, while the S-CVI for both versions was 0.99, indicating satisfactory content validity. The table summarizes the content validity index and modified kappa agreement value of both the Health-ITUES-R and the Health-ITUES-P in the validation study.

As shown in Table 3, we found a satisfactory internal consistency of the Health-ITUES-R, with Cronbach α and McDonald ω values of 0.880 and 0.899 for the overall scale, and 0.770-0.891 and 0.798-0.887 for the individual items. Similarly, the internal consistency of the Health-ITUES-P was excellent (Cronbach α=0.939 for the overall scale and 0.833-0.939 for individual items, McDonald ω=0.946 for the total scale and 0.901-0.931 for individual items). Besides, the CITC of each item in both of the versions was greater than 0.30, reflecting an acceptable correlation of each item with the sum of the other items in the scales. The table summarizes the internal consistency reliability and convergent validity of both the Health-ITUES-R and the Health-ITUES-P in the validation study.

**Figure 1:**
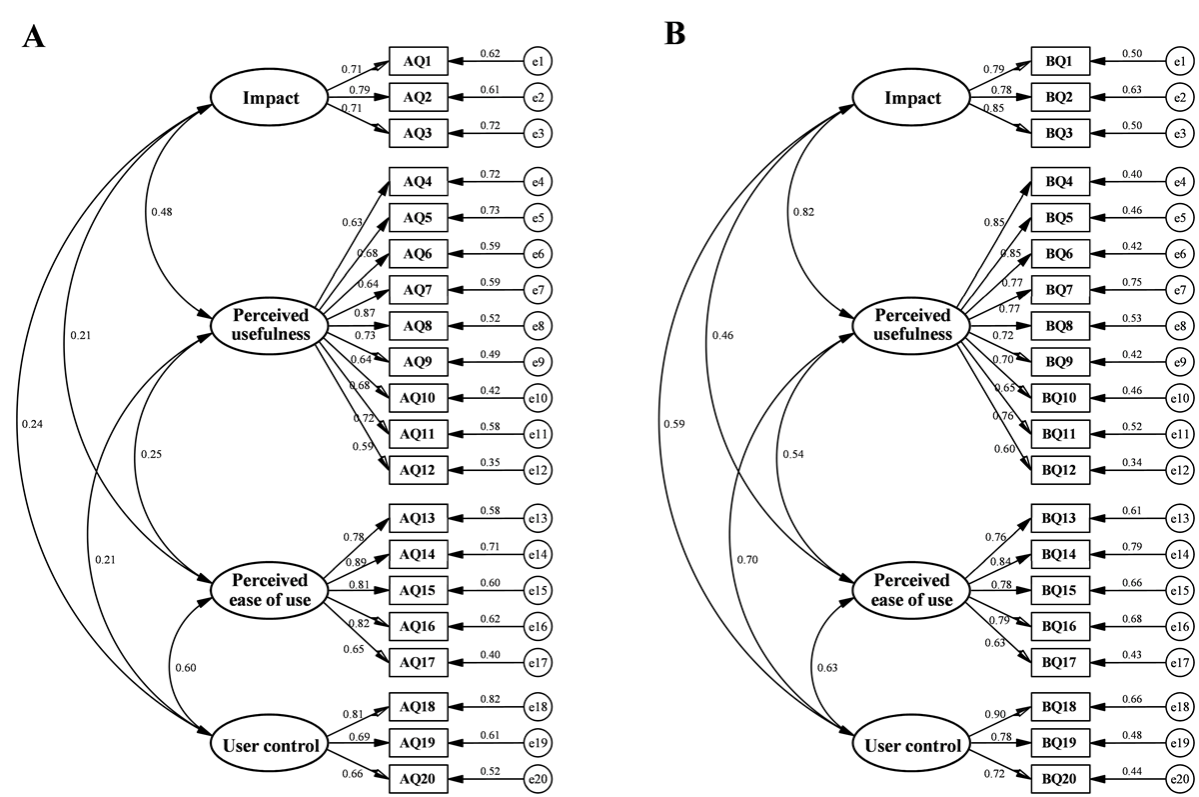
Path diagram and standardized factor loadings for both the Health-ITUES-R (A) and the Health-ITUES-P (B) in the confirmatory factor analysis. The path factor loadings were determined by critical ratios (all *P*<.001).; AQ: questions in the care receiver version; BQ: questions in the professional health care provider version; Health-ITUES-P: Health Information Technology Usability Evaluation Scale (professional health care provider version); Health-ITUES-R: Health Information Technology Usability Evaluation Scale (care receiver version).

The path diagram and standardized factor loadings of the Health-ITUES are illustrated in Figure 1. The CFA confirmed a 4-factor model consistent with the dimensions and items of the original Health-ITUES. The item scores from both the Health-ITUES-R and Health-ITUES-P exhibited adequate psychometric properties, with standardized factor loadings all exceeding 0.60, except for 1 item (AQ12) in the Health-ITUES-R (0.59). Moreover, according to the model fit indices (Table 4), both versions of the Health-ITUES showed acceptable fit, despite a slightly higher RSMEA value (0.122) for the Health-ITUES-P. The table summarizes the overall model fit of both the Health-ITUES-R and the Health-ITUES-P by using the confirmatory factor analysis in the validation study.

According to Table 3, both the Health-ITUES-R and Health-ITUES-P displayed satisfactory convergent validity, with AVE values exceeding 0.5 and CR values surpassing 0.7, except for a slightly lower AVE value (0.478) for the second dimension in the Health-ITUES-R. Besides, the greater square root of AVE values for all four dimensions than correlation coefficients and HTMT values below 0.85 suggested a good discriminant validity (Tables 5 and 6). Table 5 summarizes the discriminant validity of both the Health-ITUES-R and the Health-ITUES-P in the validation study and Table 6 summarizes the Heterotrait-Monotrait values between the 4D of both the Health-ITUES-R and the Health-ITUES-P in the validation study

Regarding the criterion validity, Pearson correlation coefficients for the perceived usefulness dimension, perceived ease of use dimension, and overall scale of the Health-ITUES-R and patient acceptance questionnaire for mobile health application were 0.587, 0.647, and 0.743, respectively (all *P*<.01), indicating a significant correlation between them. Further details are provided in Table 7.

**Table 2:** The content validity index and modified kappa agreement value of both the Health-ITUES-R^a^ and the Health-ITUES-P^b^ (n=6).

Items	Health-ITUES-R (n=6)				Health-ITUES-P (n=6)		
	Number of experts with a rating of 3 or 4	I-CVI^c^	Modified kappa	Number of experts with a rating of 3 or 4		I-CVI	Modified kappa
AQ^d^1/BQ^e^1	5	0.83	0.81	6		1.00	1.00
AQ2/BQ2	6	1.00	1.00	5		0.83	0.81
AQ3/BQ3	6	1.00	1.00	6		1.00	1.00
AQ4/BQ4	6	1.00	1.00	6		1.00	1.00
AQ5/BQ5	6	1.00	1.00	6		1.00	1.00
AQ6/BQ6	6	1.00	1.00	6		1.00	1.00
AQ7/BQ7	6	1.00	1.00	6		1.00	1.00
AQ8/BQ8	6	1.00	1.00	6		1.00	1.00
AQ9/BQ9	6	1.00	1.00	6		1.00	1.00
AQ10/BQ10	6	1.00	1.00	6		1.00	1.00
AQ11/BQ11	6	1.00	1.00	6		1.00	1.00
AQ12/BQ12	6	1.00	1.00	6		1.00	1.00
AQ13/BQ13	6	1.00	1.00	6		1.00	1.00
AQ14/BQ14	6	1.00	1.00	6		1.00	1.00
AQ15/BQ15	6	1.00	1.00	6		1.00	1.00
AQ16/BQ16	6	1.00	1.00	6		1.00	1.00
AQ17/BQ17	6	1.00	1.00	6		1.00	1.00
AQ18/BQ18	6	1.00	1.00	6		1.00	1.00
AQ19/BQ19	6	1.00	1.00	6		1.00	1.00
AQ20/BQ20	6	1.00	1.00	6		1.00	1.00

^a^Health-ITUES-R: Health Information Technology Usability Evaluation Scale (care receiver version).

^b^Health-ITUES-P: Health Information Technology Usability Evaluation Scale (professional health care provider version).

^c^I-CVI: items-level content validity index.

^d^AQ: questions in the Health Information Technology Usability Evaluation Scale (Care Receiver Version).

^e^BQ: questions in the Health Information Technology Usability Evaluation Scale (Professional Health Care Provider Version).

**Table 3:** The internal consistency reliability and convergent validity of both the Health-ITUES-R^a^ and the Health-ITUES-P^b^.

Dimensions	Health-ITUES-R						Health-ITUES-P				
	Cronbach α	McDonald ω	CITC^c^	AVE^d^	CR^e^	Cronbach α		McDonald ω	CITC	AVE	CR
Impact	0.778	0.871	—^f^	0.544	0.781	0.844		0.907	—	0.649	0.847
AQ^g^1/BQ^h^1	0.878	0.898	0.362	—	—	0.936		0.944	0.647	—	—
AQ2/BQ2	0.879	0.898	0.336	—	—	0.937		0.944	0.651	—	—
AQ3/BQ3	0.875	0.895	0.480	—	—	0.936		0.944	0.676	—	—
Perceived usefulness	0.888	0.911	—	0.478	0.891	0.914		0.931	—	0.556	0.918
AQ4/BQ4	0.873	0.894	0.523	—	—	0.935		0.943	0.722	—	—
AQ5/BQ5	0.875	0.895	0.473	—	—	0.935		0.943	0.720	—	—
AQ6/BQ6	0.877	0.896	0.416	—	—	0.936		0.943	0.697	—	—
AQ7/BQ7	0.872	0.892	0.582	—	—	0.936		0.943	0.691	—	—
AQ8/BQ8	0.875	0.894	0.486	—	—	0.936		0.944	0.647	—	—
AQ9/BQ9	0.870	0.892	0.607	—	—	0.936		0.944	0.682	—	—
AQ10/BQ10	0.875	0.895	0.482	—	—	0.935		0.943	0.694	—	—
AQ11/BQ11	0.875	0.895	0.463	—	—	0.935		0.943	0.737	—	—
AQ12/BQ12	0.875	0.895	0.493	—	—	0.937		0.945	0.609	—	—
Perceived ease of use	0.891	0.921	—	0.633	0.895	0.867		0.906	—	0.581	0.873
AQ13/BQ13	0.874	0.895	0.535	—	—	0.937		0.944	0.624	—	—
AQ14/BQ14	0.871	0.893	0.598	—	—	0.938		0.945	0.566	—	—
AQ15/BQ15	0.872	0.894	0.564	—	—	0.938		0.945	0.536	—	—
AQ16/BQ16	0.871	0.894	0.579	—	—	0.938		0.945	0.562	—	—
AQ17/BQ17	0.873	0.895	0.516	—	—	0.939		0.947	0.452	—	—
User control	0.770	0.867	—	0.527	0.768	0.833		0.901	—	0.647	0.845
AQ18/BQ18	0.874	0.895	0.494	—	—	0.936		0.943	0.696	—	—
AQ19/BQ19	0.876	0.897	0.427	—	—	0.937		0.944	0.631	—	—
AQ20/BQ20	0.879	0.900	0.314	—	—	0.937		0.945	0.596	—	—
Total scale	0.880	0.899	—	—	—	0.939		0.946	—	—	—

^a^Health-ITUES-R: Health Information Technology Usability Evaluation Scale (care receiver version).

^b^Health-ITUES-P: Health Information Technology Usability Evaluation Scale (professional health care provider version).

^c^CITC: corrected item-total correlation coefficient.

^d^AVE: average variance extracted.

^e^CR: composite reliability.

^f^Not applicable.

^g^AQ: questions in the care receiver version.

^h^BQ: questions in the professional health care provider version.

**Table 4:** Overall model fit indices of both the Health-ITUES-R^a^ and the Health-ITUES-P^b^ in the confirmatory factor analysis.

Model fit indices	χ^2^/df ^c^	RMSEA^d^	RMR^e^	PGFI^f^	PNFI^g^	PCFI^h^	SRMR^i^
Observed Value of the Health-ITUES-R	1.621	0.075	0.031	0.629	0.676	0.778	0.076
Observed Value of the Health-ITUES-P	2.841	0.122	0.033	0.571	0.652	0.711	0.079
Level of acceptance	<3	≤0.10	≤0.05	≥0.5	≥0.5	≥0.5	≤0.1

^a^Health-ITUES-R: Health Information Technology Usability Evaluation Scale (care receiver version).

^b^Health-ITUES-P: Health Information Technology Usability Evaluation Scale (professional health care provider version).

^c^χ^2^/*df*: the ratio of χ^2^ to *df*.

^d^RMSEA: root-mean-square error of approximation.

^e^RMR: root-mean-square residual.

^f^PGFI: parsimonious goodness-of-fit index.

^g^PNFI: parsimonious normed fit index.

^h^PCFI: parsimony comparative fit index.

^i^SRMR: standardized root-mean-square residual.

**Table 5:** Discriminant validity of both the Health-ITUES-R^a^ and the Health-ITUES-P^b^,^c^.

Dimensions	Health-ITUES-R					Health-ITUES-P			
	D1^d^	D2^e^	D3^f^	D4^g^	D1		D2	D3	D4
D1	0.738	—^h^	—	—	0.806		—	—	—
D2	0.484	0.691	—	—	0.747		0.746	—	—
D3	0.215	0.248	0.796	—	0.461		0.545	0.762	—
D4	0.237	0.210	0.602	0.726	0.594		0.702	0.630	0.804

^a^Health-ITUES-R, Health Information Technology Usability Evaluation Scale (care receiver version).

^b^Health-ITUES-P, Health Information Technology Usability Evaluation Scale (professional health care provider version).

^c^The values on the diagonal are the square root of average variance extracted.

^d^D1: dimension 1 (impact).

^e^D2: dimension 2 (perceived usefulness).

^f^D3: dimension 3 (perceived ease of use).

^g^D4: dimension 4 (user control).

^h^Not applicable.

**Table 6:** HTMT^a^ values between the 4D of both the Health-ITUES-R^b^ and the Health-ITUES-P^c^.

HTMT values	Health-ITUES-R	Health-ITUES-P
Impact-perceived usefulness	0.506	0.845
Impact-perceived ease of use	0.240	0.463
Impact-user control	0.262	0.604
Perceived usefulness-perceived ease of use	0.281	0.589
Perceived usefulness-user control	0.214	0.753
Perceived ease of use-user control	0.631	0.687

^a^HTMT: Heterotrait-Monotrait.

^b^Health-ITUES-R: Health Information Technology Usability Evaluation Scale (care receiver version).

^c^Health-ITUES-P: Health Information Technology Usability Evaluation Scale (professional health care provider version).

**Table 7:** Criterion validity of the Health-ITUES-R^a^ concerning the patient acceptance questionnaire for mobile health app.

Health-ITUES-R	Patient acceptance questionnaire for mobile health app						
	Usefulness	Ease of use	System or interface	Reliability	Usage attitude	Usage tendency	Overall scale
Impact	0.383^b^	0.181	0.288^b^	0.275^b^	0.323^b^	0.317^b^	0.389^b^
Perceived usefulness	0.587^b^	0.222^c^	0.320^b^	0.401^b^	0.531^b^	0.492^b^	0.563^b^
Perceived ease of use	0.453^b^	0.647^b^	0.417^b^	0.087	0.492^b^	0.393^b^	0.576^b^
User control	0.456^b^	0.406^b^	0.310^b^	0.107	0.368^b^	0.335^b^	0.452^b^
Overall scale	0.696^b^	0.546^b^	0.488^b^	0.330^b^	0.657^b^	0.580^b^	0.743^b^

^a^Health-ITUES-R: Health Information Technology Usability Evaluation Scale (Care Receiver Version).

^b^*P*<.01.

^c^*P*<.05.

## Discussion

### Principal Findings

Based on the original Health-ITUES, we meticulously translated and culturally adapted it to develop the Chinese version of the Health-ITUES. The validation test conducted among the main users of the SMART system (older people and nurses) confirmed satisfactory reliability and validity of the Chinese version of the Health-ITUES in evaluating the usability of digital health apps. To our understanding, this represents the first exploration of a valid usability evaluation instrument specifically designed for digital health apps considering both care receivers and professional health care providers in China, which can provide evidence supporting the use of the Chinese version of the Health-ITUES as a validated tool for evaluating the usability of digital health apps.

Following the guidelines for the process of cross-cultural adaptation of self-report measures [20], we carefully selected appropriate translators for both forward and back translations of the Health-ITUES and determined the Chinese version through numerous rounds of discussions within the research team [40]. Simultaneously, our iterative modification process under consultations with the expert panel until obtaining verification of the original author, enabled us to adjust the dimensions and items from a professional perspective and ensure that the original meanings of the Health-ITUES items were retained, thereby improving the effectiveness and practicality of the Chinese version of the Health-ITUES [41].

Furthermore, the customized Health-ITUES-R and Health-ITUES-P were validated as effective tools with good content validity, internal consistency reliability, and discriminant validity in measuring the usability of the SMART system. The CFA results also indicated an adequate structure validity, except for a slightly higher RMSEA value of the Health-ITUES-P. It is worth noting that the RMSEA value is calculated based on non-centrality parameters for representing the absolute measure of fit, and its calculation heavily depends on the sample size [42,43]. Models with smaller sample sizes were generally believed with the potential to artificially large RMSEA values, which can explain the overestimated RMSEA value in our validation test to some extent [44]. Regarding the convergent validity, the slightly lower AVE value for the second dimension in the Health-ITUES-R (0.478) could be attributed to the limited understanding and short usage time of the SMART system among older individuals. This may result in less precise responses to the 9 questions in this dimension and a lower AVE value [45]. In addition, the Health-ITUES-R exhibited high criterion validity compared to the patient acceptance questionnaire for mobile health apps.

To the best of our knowledge, our study, for the first time, formulated the Chinese version of the Health-ITUES and validated its utility for evaluating the usability of digital health apps in the Chinese context by considering both care receivers and professional health care providers [8]. In addition to the commonly used validation measures such as content validity, internal consistency reliability, structure validity, and criterion validity, we also examined the convergent and discriminant validity for a comprehensive validation of the Chinese version of the Health-ITUES. Given its strong psychometric properties, we postulate that the Chinese version of the Health-ITUES can serve as a valuable instrument in evaluating the usability of digital health apps for both professional health care providers and receivers.

### Limitations

There are several limitations in this study. First, the validation of the Chinese version of the Health-ITUES relied on the SMART system, a platform designed for personalized integrated home-based older care, while the Health-ITUES applies to all types of digital health apps. Consequently, it cannot be ruled out that the validation results could have differed with another app. Second, there may be a selection bias in the sample selection, since the validation tests were conducted in the geriatric ward of a comprehensive hospital, where individuals tended to spend more time using digital health apps to manage their health status compared to the general population [46]. Furthermore, our validation study was constrained by relatively small sample sizes and inadequate usage time of the SMART system due to the COVID-19 pandemic. The generalizability of the findings should be approached with caution. Further research with a larger sample and adequate usage time of the digital health apps is needed.

### Conclusions

This study formulated a Chinese version of the Health-ITUES with satisfactory reliability and validity in evaluating the usability of the digital health apps for both care receivers and professional health care providers. The Chinese version of the Health-ITUES can serve as a valuable tool to identify reliable and effective digital health apps for end users. Future research focusing on the validation of the Health-ITUES in diverse cultural contexts and settings is crucial for enhancing its applicability and effectiveness across different populations.

## Appendix

Multimedia Appendix 1 Additional methods and results.

Multimedia Appendix 2 Reporting Guideline Checklist.

## Abbreviations

AVE: average variance extracted
Ca: judgment coefficient
CFA: confirmatory factor analysis
CITC: corrected item-total correlation coefficient
Cr: authority coefficient
CR: composite reliability
Cs: familiarity coefficient
CSUQ: Computer System Usability Questionnaire
EFA: exploratory factor analysis
Health-ITUES: Health Information Technology Usability Evaluation Scale
Health-ITUES-P: Health Information Technology Usability Evaluation Scale—professional health care provider version
Health-ITUES-R: Health Information Technology Usability Evaluation Scale—care receiver version
HTMT: Heterotrait-Monotrait ratio
I-CVI: item-level content validity index
PCFI: parsimonious comparative fit index
PGFI: parsimonious goodness-of-fit index
PNFI: parsimonious normed fit index
PSSUQ: Post-Study System Usability Questionnaire
RMR: root-mean-square residual
RMSEA: root-mean-square error of approximation
S-CVI: scale-content validity index
SRMR: standardized root-mean-square residual
STROBE: Strengthening the Reporting of Observational Studies in Epidemiology
SUS: System Usability Scale

## Data Availability

The datasets generated or analyzed during this study are not publicly available due to ethical and legal restrictions but are available from the corresponding author on reasonable request.
