# Supplementary material for: Translation, Cross-Cultural Adaptation, and Psychometric Validation of the Health Information Technology Usability Evaluation Scale in China: Instrument Validation Study
Source: J Med Internet Res. 2025 May 2;27:e67948. doi: 10.2196/67948 (PMC12084768; doi:10.2196/67948)
Supplement: Multimedia Appendix 1 [file jmir_v27i1e67948_app1.docx]

# **The Health Information Technology Usability Evaluation Scale: Translation, cross-cultural adaptation, and psychometric validation in China**

CONTENTS

Figure S1 The permission from the original author of the Health Information Technology Usability Evaluation Scale2

Table S1. Basic information of the translators involved in the forward translation, synthesis of the forward translations, and back translation3

Table S2. Basic information of the expert panel for cross-cultural adaption4

Table S3. Methods and rules to assess the expert authority coefficient5

Table S4. Basic information of the experts involved in the customization of the Health-ITUES-R and Health-ITUES-P6

Table S5. The specific Aifuxing Application operation tasks assigned to the older individuals and nurses during the validation test7

Table S6. The original version, forward translation versions, synthesized version, and back translation versions of the Health-ITUES8

Table S7. The expert suggestions and specific modifications from the first round of expert consultation of the synthesized forward translation version T312

Table S8. The expert suggestions and specific modifications from the second round of expert consultation of the synthesized forward translation version T314

Table S9. The expert suggestions and specific modifications of the initial Health-ITUES-R15

Table S10. The expert suggestions and specific modifications of the initial Health-ITUES-P17

Table S11. The finalized customized Health-ITUES-R18

Table S12. The finalized customized Health-ITUES-P 20

Table S13. Path and standardized factor loadings of the Health-ITUES-R22

Table S14. Path and standardized factor loadings of the Health-ITUES-P23

Table S15 Patient Acceptance Questionnaire for Mobile Health Application24

Figure S1 The permission from the original author of the Health Information Technology Usability Evaluation Scale


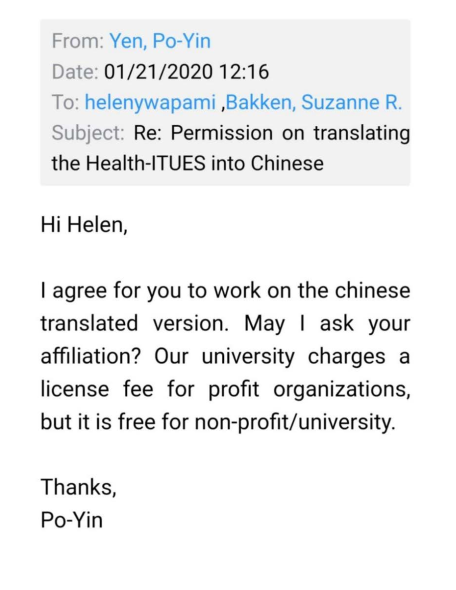


Table S1. Basic information of the translators involved in the forward translation, synthesis of the forward translations, and back translation

| No. | Educational background | Professional title | Research field | Awareness of the scale | Translation work |
| --- | --- | --- | --- | --- | --- |
| 1 | Postgraduate degree | Associate professor | Older care  Smart care | No | Forward translation |
| 2 | Postgraduate student | None | Nursing information  Smart care | Yes | Forward translation |
| 3 | Postgraduate degree | Associate professor | Nursing information | Yes | Synthesis of the forward translations |
| 4 | Postgraduate degree | Associate professor | Medicine | No | Back translation |
| 5 | Postgraduate degree | Professor | Medicine | No | Back translation |

The table summarizes the basic information of the involved translators, including their educational background, professional title, research field, awareness of the scale, and the translation tasks they are responsible for.

Table S2. Basic information of the expert panel for cross-cultural adaption

| No. | Educational background | Professional title | Research field | Years of employment |
| --- | --- | --- | --- | --- |
| 1 | Postgraduate degree | Professor | Older welfare technology  Data Science and Engineering | 19 |
| 2 | Bachelor degree | Associate chief nurse | Clinical Nursing | 29 |
| 3 | Postgraduate degree | Associate professor | Nursing Information | 8 |
| 4 | Postgraduate degree | Assistant professor | Chinese and American Culture  Interpersonal communication  Psychology | 15 |
| 5 | Postgraduate degree | Associate professor | Older care  Smart care | 16 |
| 6 | Postgraduate degree | Associate professor | International Comparative Education in Health Sciences | 8 |

The table summarizes the basic information of the experts involved in the cross-cultural adaption, including their educational background, professional title, research field, and the employment years.

Table S3. Methods and rules to assess the expert authority coefficient

| Judgment coefficient | The degree to which experts are affected | | | Familiarity coefficient | |
| --- | --- | --- | --- | --- | --- |
| (Ca) | Major | Moderate | Minor | (Cs) | |
| Practical experience | 0.5 | 0.4 | 0.3 | Very familiar | 1.0 |
| Theoretical analysis | 0.3 | 0.2 | 0.1 | Familiar | 0.8 |
| References | 0.1 | 0.1 | 0.1 | Average | 0.6 |
| Intuitive feeling | 0.1 | 0.1 | 0.1 | Not very familiar | 0.4 |
|  |  |  |  | Unfamiliar | 0.2 |

The table summarizes the methods and rules to assess the expert authority coefficient during the cross-cultural adaption.

Table S4. Basic information of the experts involved in the customization of the Health-ITUES-R and Health-ITUES-P

| No. | Educational background | Professional title | Research field | Years of employment | Participation in translation |
| --- | --- | --- | --- | --- | --- |
| 1 | Postgraduate degree | Chief nurse | Nursing information  Nurse quality | 29 | No |
| 2 | Postgraduate degree | Professor | Nursing Information  Chronic disease management | 28 | No |
| 3 | Postgraduate degree | Professor | Nursing management  Nursing information | 23 | No |
| 4 | Postgraduate degree | Professor | Older welfare technology  Data Science and Engineering | 19 | Yes |
| 5 | Postgraduate degree | Associate professor | Nursing Information | 8 | Yes |
| 6 | Postgraduate degree | Assistant professor | Chinese and American Culture  Psychology | 15 | Yes |

The table summarizes the basic information of the experts involved in the customization of both the Health-ITUES-R and the Health-ITUES-P, including their educational background, professional title, research field, employment years, and whether participated in the proit translation or not; Health-ITUES-R, Health Information Technology Usability Evaluation Scale (Care Receiver Version); Health-ITUES-P, Health Information Technology Usability Evaluation Scale (Professional Care Provider Version).

Table S5. The specific Aifuxing Application operation tasks assigned to the older individuals and nurses during the validation test

|  | Older individuals | Nurses |
| --- | --- | --- |
| Task 1 | To view health reports independently at least once | To conduct a health assessment for older adults independently at least once |
| Task 2 | To input medication information independently at least once | To view health reports independently at least once |
| Task 3 | To record blood sugar or blood pressure independently at least once | To input the older people’s blood sugar or blood pressure independently at least once |
| Task 4 | To record diet or exercise independently at least once | To check the personalized interventions independently at least once |
| Task 5 | To access health information independently at least once | To edit temporary notifications independently at least once |

Table S6. The original version, forward translation versions, synthesized version, and back translation versions of the Health-ITUES

|  | Original version | T1 | T2 | T3 | BT1 | BT2 |
| --- | --- | --- | --- | --- | --- | --- |
| Dimension 1 | Quality of Work Life | Work quality | Quality of Work Life | Quality of Work Life | Quality of Work Life | Work and Life Quality |
| Q1 | I think [BidShift] has been [a positive addition to Nursing]. | I think [BidShift] has a positive effect on nursing. | I think [BidShift] has a positive effect on nursing. | I think [BidShift] has a positive effect on nursing. | I think [Bidshift] has a positive effect on nursing. | I think that [Bidshift] has a positive effect on nursing. |
| Q2 | I think BidShift has been [a positive addition to our organization]. | I think [BidShift] has a positive effect on our organization and work. | I think [BidShift] has a positive effect on our organization. | I think [BidShift] has a positive effect on our organizational work. | I think [Bidshift] has a positive effect on our organizational tasks. | I think that [Bidshift] has a positive effect on our organization. |
| Q3 | [BidShift technology] is an important part of [our staffing process]. | The [Bidshift] is an important component of our staffing process. | The [Bidshift] is an important part of our staffing process. | [Bidshift] is an important part of our staffing process. | [Bidshift] is an important part of the staffing process. | [Bidshift] is an important part of the staffing process. |
| Dimension 2 | Perceived Usefulness | Perceived Usefulness | Perceived Usefulness | Perceived Usefulness | Perceived Usefulness | Perceived Usefulness |
| Q4 | Using [Bidshift] makes it easier to [request the shift I want]. | Using [Bidshift] makes it easier to get the work I want. | Using [Bidshift] makes it easier to request the shift I want. | Using [Bidshift] makes it easier to apply for the shift I want. | Using [Bidshift] makes it easier for me to request the shift I prefer/want. | Using [Bidshift] makes it easier for me to apply for the shift that I want. |
| Q5 | Using [Bidshift] enables me to [request shifts] more quickly. | Using [Bidshift] makes me get the work more quickly. | Using [Bidshift] enables me to apply for the shift more quickly. | Using [Bidshift] enables me to request shifts more quickly. | Using [Bidshift] allows/enables me to request shifts more quickly. | Using [Bitshift] allows me to apply for shifts more quickly. |
| Q6 | Using [Bidshift] makes it more likely that I [will be awarded a shift that I request]. | Using [Bidshift] makes it more possible for me to complete my work. | Using [Bidshift] makes it more likely to get a shift that I request. | Using [Bidshift] makes it more likely that I will get a shift that I apply for. | Using [Bidshift] makes it more likely for me to  acquire/get the shift I apply for/request. | Using [Bidshift] makes it more possible for me to get the shifts that I applied for. |
| Q7 | Using [Bidshift] is useful for [requesting open shifts]. | Using [Bidshift] is useful for me to apply for open works. | Using [Bidshift] is useful for requesting open shifts. | Using [Bidshift] is useful for requesting open shifts. | Utilizing/using [Bidshift] is useful for requesting open shifts. | Using [Bidshift] is useful for applying for open shifts. |
| Q8 | I think [Bidshift] presents a more equitable process for [requesting open shifts]. | I think [Bidshift] provides a more reasonable process to apply for open work. | I think [Bidshift] provides a fairer process to request open shifts. | I think [Bidshift] provides a more reasonable process to request open shifts. | I think [Bidshift] offers a more reasonable process for requesting open shifts. | I think that [Bidshift]  provides a more systematic process for applying to open shifts. |
| Q9 | I am satisfied with [Bidshift] for [requesting open shifts]. | I am very satisfied with the use of [Bidshift] to require open work. | I am very satisfied with using [Bidshift] to apply for open shifts. | I am very satisfied with using [Bidshift] to request open shifts. | I am satisfied with using [Bidshift] to request open shifts. | I am very satisfied with using [Bidshift] to apply for open shifts. |
| Q10 | I [am awarded shifts] in a timely manner because of [Bidshift]. | Using [Bidshift] enables me to finish my work on time. | Due to [Bidshift], I can obtain shifts promptly. | Because of [Bidshift], I can obtain shifts in a timely manner. | I could find shifts in a timely manner because  of [Bidshift]. | Because of [Bidshift], I can get shifts in time. |
| Q11 | Using [Bidshift] increases [requesting open shifts]. | Using [Bidshift] can increase requiring open work. | Use [Bidshift] promotes the application for open shifts. | Using [Bidshift] promotes requesting open shifts. | Using [Bidshift] could boost/increase requesting open shifts. | Using [Bidshift] can facilitate applying for open shifts. |
| Q12 | I am able to [find shifts that I am qualified to work] whenever I use [Bidshift]. | I can complete the work I need to do whenever I use familiar functions of the [Bidshift]. | As long as I use [Bidshift], I can find the shift that suits my work. | I can find shifts that suit my work well whenever I use [Bidshift]. | Whenever I use [Bidshift], I am/was able to find the shifts that suit me. | Anytime that I use [Bidshift], I always find a shift that best fits my job. |
| Dimension 3 | Perceived Ease of Use | Perceived Ease of Use | Perceived Ease of Use | Perceived Ease of Use | Perceived Ease of Use | Perceived Ease of Use |
| Q13 | I am comfortable with my ability to use [Bidshift]. | I feel very comfortable using [Bidshift]. | I am satisfied with my ability to use [Bidshift]. | I am comfortable with my ability of using [Bidshift]. | I am satisfied/comfortable with my ability to use  [Bidshift]. | I am satisfied with my ability to use [Bidshift]. |
| Q14 | Learning to operate [Bidshift] is easy for me. | I can easily learn to operate [Bidshift]. | Learning to operate [Bidshift] is very easy for me. | Learning to use Bidshift is very easy for me. | Learning to use [Bidshift] is/was easy for me. | Learning how to use [Bidshift] was easy for me. |
| Q15 | It is easy for me to become skillful at using [Bidshift]. | I can easily become proficient in using [Bidshift]. | It is easy for me to become proficient in using [Bidshift]. | It is easy for me to become proficient in using [Bidshift]. | It is/was easy for me to become skillful at using [Bidshift]. | I am skilled at using [Bidshift]. |
| Q16 | I find [Bidshift] easy to use. | I find [Bidshift] very easy to use. | I find that [Bidshift] is very easy to use. | I find [Bidshift] very easy to use. | I find/found [Bidshift]  easy to use. | I find that [Bidshift] is easy to use. |
| Q17 | I can always remember how to log on to and use [Bidshift]. | I always easily remember how to log on and use [Bidshift]. | I can always remember how to log in and use [Bidshift]. | I can always remember how to log on to and use [Bidshift]. | I could always remember how to log on to and use [Bidshift]. | I always remember how to log in and use  [Bidshift]. |
| Dimension 4 | User Control | User Control | User Control | User Control | User Support | User Control |
| Q18 | [Bidshift] gives error messages that clearly tell me how to fix problems. | [Bidshift] can provide clear error information and tell me how to solve the problems. | [Bidshift] can provide error information to clearly tell me how to fix the problems. | [Bidshift] can provide error information that clearly tells me how to fix the problems. | [Bidshift] sends error massages that clearly tell me how to fix the problems. | [Bidshift] provides clear error messages that I can easily follow to solve problems. |
| Q19 | Whenever I make a mistake using [Bidshift], I recover easily and quickly. | Whenever there is a usage error with [Bidshift], I can easily and quickly restore it to normal. | Whenever there is an error during the use of [Bidshift], I can easily and quickly recover. | Whenever I make an error during the use of [Bidshift], I can easily and quickly recover. | Whenever I made a  mistake using [Bidshift], I could recover easily and quickly. | Whenever I make a mistake while using  [Bidshift], I can quickly resolve it. |
| Q20 | The information (such as on-line help, on-screen messages and other documentation) provided with [Bidshift] is clear. | The information provided by [Bidshift], such as online help, screen information, and other document information, is clear. | The information provided by Bidshift, such as online help, screen messages, and other documents, is clear. | The information provided by Bidshift (such as online help, screen information, and other document information) is clear. | The information (e.g. online help, onscreen messages, and other  documentation) provided by [Bidshift] is clear. | Information provided by [Bidshift] (e.g., online help, screen information, and other documentation) is clear. |

The table summarizes the original version, two forward translation versions, one synthesized version, and two back translation versions of the Health-ITUES; T1 and T2, forward translation versions; T3, synthesized version; BT1 and BT2, back translation versions; Health-ITUES, Health Information Technology Usability Evaluation Scale.

Table S7. The expert suggestions and specific modifications from the first round of expert consultation of the synthesized forward translation version T3

| Dimensions/  Items | Contents of the synthesized version T3 | Expert suggestions | Modifications |
| --- | --- | --- | --- |
| Q2 | I think [BidShift] has a positive effect on our organizational work. | Two experts suggested translating “organizational work” into “organization” because “organization” is more commonly used. | We accepted the suggestion and modified this item to “I think [BidShift] has a positive effect on our organization”. |
| Dimension 2 | Perceived Usefulness | One expert expressed confusion and misunderstanding about this dimension. | No modification was made.  Reason: “Perceived Usefulness” is a core part in technology acceptance model, which has been widely used in China. |
| Q8 | I think [Bidshift] provides a more reasonable process to request open shifts. | Three experts advised that the term “reasonable” should be translated as “fair”. | We accepted the suggestion and modified this item to “I think [Bidshift] provides a more equitable process to request open shifts”. |
| Q9 | I am very satisfied with using [Bidshift] to request open shifts. | Two experts suggested removing “very” to better align with expression habits. | We accepted the suggestion and modified this item to “I am satisfied with using [Bidshift] to request open shifts”. |
| Q10 | Because of [Bidshift], I can obtain shifts in a timely manner. | One expert suggested replacing “because of” with “due to” for a more concise expression. | No modification was made.  Reason: Considering speaking habits, “because of” is easier to understand. |
| Q11 | Using [Bidshift] promotes requesting open shifts. | Three experts suggested replacing “promote” with “increase” for a clearer meaning. | We accepted the suggestion and modified this item to “Using [Bidshift] increases requesting open shifts”. |
| Q12 | I can find shifts that suit my work well whenever I use [Bidshift]. | Two experts pointed out that “suit” should be changed into “qualified”, and an expert suggested changing “whenever” to “as long as”. | We accepted the suggestions and modified this item to “As long as I use [Bidshift], I can find shifts that I am qualified”. |
| Dimension 3 | Perceived ease of use | One expert felt confused about this dimension. | No modification was made.  Reason: “Perceived ease of use” is a core part in the technology acceptance model, which has been widely used in China. |
| Q16 | I find [Bidshift] very easy to use. | One expert suggested translating “find” as “feel”. | No modification was made.  Reason: The Chinese meaning of “find” does not include “fell”, and the word “find” could better reflect what users conclude after repeated thinking. |
| Dimension 4 | User control | One expert advised translating the dimension as “User Control Ability”. | We accepted the suggestion and modified this item to “User Control Ability”. |

The table summarizes the expert suggestions and specific modifications from the first round of expert consultation of the synthesized forward translation version of the Health-ITUES.

Table S8. The expert suggestions and specific modifications from the second round of expert consultation of the synthesized forward translation version T3

| Dimensions/  Items | Contents of the synthesized version T3 | Expert suggestions | Modifications |
| --- | --- | --- | --- |
| Dimension 1 | Quality of Work Life | One expert suggested translating the dimension into “impact” because some items in this dimension do not belong to “quality of work life”. | We accepted the suggestion and modified this dimension to “impact”. |
| Q2 | I think [BidShift] has a positive effect on our organization. | One expert pointed out that “organization” in English is sometimes equivalent to “unit” in Chinese. | We accepted the suggestion and modified this item to “I think [BidShift] has a positive effect on our unit”. |
| Q11 | Using [Bidshift] increases requesting open shifts. | Two experts suggested that “request open shifts” could be changed to “application of open shifts”, which is more in line with speaking habits. | We accepted the suggestion and modified this item to “Using [Bidshift] increases application of open shifts”. |
| Q12 | As long as I use [Bidshift], I can find shifts that I am qualified. | An expert pointed out that “as long as” is equal to “only if” in English and does not reflect the original meaning of “whenever”. | We accepted the suggestion and modified this item to “Whenever I use [Bidshift], I can find shifts that I am qualified”. |
| Dimension 4 | User Control Ability | One expert believed that “User Control” is more effective in expressing the original meaning than “User Control Ability”. | We modified this dimension to “user control” following suggestions from the author of the original scale. |

The table summarizes the expert suggestions and specific modifications from the second round of expert consultation of the synthesized forward translation version of the Health-ITUES.

Table S9. The expert suggestions and specific modifications of the initial Health-ITUES-R

| Dimensions/  Items | Contents | Expert suggestions | Modifications |
| --- | --- | --- | --- |
| Dimension 1 | Impact | Experts suggested adding a modifier before “impact” to make it clearer, or changing it to “quality of life”. | No modification was made.  Reason: Some items in this dimension do not belong to the “quality of life”. |
| AQ1 | I think the Aifuxing App has a positive effect on home-based care services. | Experts advised changing “home-based care services” to “obtain home-based care services”. | We accepted the suggestion and modified this item to “I think the Aifuxing App has a positive effect on obtaining home-based care services”. |
| AQ2 | I think the Aifuxing App can improve older people’s quality of life. | Experts suggested changing “older people’s quality of life” to “my quality of life” based on “our organization” in the original scale. | We accepted the suggestion and modified this item to “I think the Aifuxing App can improve my quality of life”. |
| AQ4 | Using the Aifuxing App makes it easier for me to apply for home-based care services. | Experts believed that adding “that I need” after “home-based care services” is more specific. | We accepted the suggestion and modified this item to “Using the Aifuxing App makes it easier for me to apply for home-based care services that I need”. |
| AQ6 | Using the Aifuxing App makes it more likely to obtain the home-based care services. | Experts recommended adding “that I need” after “home-based care services”. | We accepted the second suggestion and modified this item to “Using the Aifuxing App makes it more likely to obtain the home-based care services that I need”. |
| AQ8 | I think the Aifuxing App provides a more equitable process to request home-based care services. | Experts proposed a need to replace “process” with “choice”. | We accepted the second suggestion and modified this item to “I think the Aifuxing App provides a more equitable choice to request home-based care services”. |
| AQ11 | Using the Aifuxing App promotes the application of home-based care services. | Experts suggested adding the subject “I”. | No modification was made.  Reason: The research team unanimously agreed that not adding a subject did not affect the sentence's meaning. |
| AQ14 | Learning to use the Aifuxing App is easy for me. | Experts pointed out that AQ14 and AQ15 may confuse respondents. | We accepted the suggestion and modified this item to “Learning how to use the Aifuxing App is easy for me”. |
| AQ20 | The information (such as online help, on-screen messages, and other documentation) provided by the Aifuxing App is clear. | Experts recommended making appropriate modifications to this item based on specific functions of the Aifuxing App. | No modification was made.  Reason: The original content is more applicable to measuring older people’s perceived usability of the Aifuxing App. |

The table summarizes the expert suggestions and specific modifications of the initial Health-ITUES-R; Health-ITUES-R, Health Information Technology Usability Evaluation Scale (Care Receiver Version); App, application; AQ, questions in the Care Receiver Version.

Table S10. The expert suggestions and specific modifications of the initial Health-ITUES-P

| Dimensions/  Items | Contents | Expert suggestions | Modifications |
| --- | --- | --- | --- |
| BQ1 | I think the Aifuxing App has a positive effect on home-based care services. | Experts suggested adding “provide” before “home-based care services”. | We accepted the suggestion and modified this item to “I think the Aifuxing App has a positive effect on providing home-based care services”. |
| BQ2 | I think the Aifuxing App has a positive effect on providing home-based care services for my unit. | Experts recommended changing “my unit” to “our unit”. | We accepted the suggestion and modified this item to “I think the Aifuxing App has a positive effect on providing home-based care services for my unit”. |
| BQ6 | Using the Aifuxing App makes it more likely to provide personalized home-based care services. | Experts expressed confusion about the term “personalized”. | No modification was made.  Reason: The Aifuxing App allowed professional healthcare providers to offer personalized care interventions for older people according to their heterogeneous characteristics and specific care problems. |
| BQ8 | I think the Aifuxing App provides a more equitable process to provide home-based care services. | Experts proposed a need to replace “process” with “choice”. | We accepted the suggestion and modified this item to “I think the Aifuxing App provides a more equitable choice to provide home-based care services”. |
| BQ12 | I can provide personalized home-based care services for older people whenever I use the Aifuxing App. | Experts suggested adding “for older people” for more specific expression. | We accepted the suggestion and modified this item to “I can provide personalized home-based care services for older people whenever I use the Aifuxing App”. |

The table summarizes the expert suggestions and specific modifications of the initial Health-ITUES-P; Health-ITUES-P, Health Information Technology Usability Evaluation Scale (Professional Care Provider Version); App, application; BQ, questions in the Professional Care Provider Version.

Table S11. The finalized customized Health-ITUES-R

| Content | Degree | | | | |
| --- | --- | --- | --- | --- | --- |
| Impact | | | | | |
| 1. I think the Aifuxing App has a positive effect on obtaining home-based care services. | Completely disagree | Disagree | Neutral | Agree | Completely agree |
| 2. I think the Aifuxing App can improve my quality of life. | Completely disagree | Disagree | Neutral | Agree | Completely agree |
| 3. The Aifuxing App is an important tool for me to access home-based care services. | Completely disagree | Disagree | Neutral | Agree | Completely agree |
| Perceived usefulness | | | | | |
| 4. Using the Aifuxing App makes it easier for me to apply for home-based care services that I need. | Completely disagree | Disagree | Neutral | Agree | Completely agree |
| 5. Using the Aifuxing App enables me to apply for home-based care services more quickly. | Completely disagree | Disagree | Neutral | Agree | Completely agree |
| 6. Using the Aifuxing App makes it more likely to obtain the home-based care services that I need. | Completely disagree | Disagree | Neutral | Agree | Completely agree |
| 7. Using the Aifuxing App is useful for me to apply for home-based care services. | Completely disagree | Disagree | Neutral | Agree | Completely agree |
| 8. I think the Aifuxing App provides a more equitable choice to request home-based care services. | Completely disagree | Disagree | Neutral | Agree | Completely agree |
| 9. I am satisfied with the Aifuxing App for applying home-based care services. | Completely disagree | Disagree | Neutral | Agree | Completely agree |
| 10. The Aifuxing App allows me to obtain home-based care services promptly. | Completely disagree | Disagree | Neutral | Agree | Completely agree |
| 11. Using the Aifuxing App promotes the application of home-based care services. | Completely disagree | Disagree | Neutral | Agree | Completely agree |
| 12. I can obtain home-based care services that I need whenever I use the Aifuxing App. | Completely disagree | Disagree | Neutral | Agree | Completely agree |
| Perceived ease of use | | | | | |
| 13. I am comfortable with my ability to use the Aifuxing App. | Completely disagree | Disagree | Neutral | Agree | Completely agree |
| 14. Learning how to use the Aifuxing App is easy for me. | Completely disagree | Disagree | Neutral | Agree | Completely agree |
| 15. I can easily become skillful at using the Aifuxing App. | Completely disagree | Disagree | Neutral | Agree | Completely agree |
| 16. I find the Aifuxing App easy to use. | Completely disagree | Disagree | Neutral | Agree | Completely agree |
| 17. I can always remember how to log on to and use the Aifuxing App. | Completely disagree | Disagree | Neutral | Agree | Completely agree |
| User control | | | | | |
| 18. The Aifuxing App gives messages that tell me how to fix problems. | Completely disagree | Disagree | Neutral | Agree | Completely agree |
| 19. Whenever I make a mistake using the Aifuxing App, I recover easily and quickly. | Completely disagree | Disagree | Neutral | Agree | Completely agree |
| 20. The information (such as online help, on-screen messages, and other documentation) provided by the Aifuxing App is clear. | Completely disagree | Disagree | Neutral | Agree | Completely agree |

Health-ITUES-R, Health Information Technology Usability Evaluation Scale (Care Receiver Version); App, application.

Table S12. The finalized customized Health-ITUES-P

| Content | Degree | | | | |
| --- | --- | --- | --- | --- | --- |
| Impact | | | | | |
| 1. I think the Aifuxing App has a positive effect on providing home-based care services. | Completely disagree | Disagree | Neutral | Agree | Completely agree |
| 2. I think the Aifuxing App has a positive effect on providing home-based care services for my unit. | Completely disagree | Disagree | Neutral | Agree | Completely agree |
| 3. The Aifuxing App is an important tool for me to provide home-based care services. | Completely disagree | Disagree | Neutral | Agree | Completely agree |
| Perceived usefulness | | | | | |
| 4. Using the Aifuxing App makes it easier for me to provide home-based care services. | Completely disagree | Disagree | Neutral | Agree | Completely agree |
| 5. Using the Aifuxing App enables me to provide home-based care services more quickly. | Completely disagree | Disagree | Neutral | Agree | Completely agree |
| 6. Using the Aifuxing App makes it more likely to provide personalized home-based care services. | Completely disagree | Disagree | Neutral | Agree | Completely agree |
| 7. Using the Aifuxing App is useful for me to provide home-based care services. | Completely disagree | Disagree | Neutral | Agree | Completely agree |
| 8. I think the Aifuxing App provides a more equitable choice to provide home-based care services. | Completely disagree | Disagree | Neutral | Agree | Completely agree |
| 9. I am satisfied with the Aifuxing App for providing home-based care services. | Completely disagree | Disagree | Neutral | Agree | Completely agree |
| 10. The Aifuxing App allows me to provide home-based care services promptly. | Completely disagree | Disagree | Neutral | Agree | Completely agree |
| 11. Using the Aifuxing App promotes the provision of home-based care services. | Completely disagree | Disagree | Neutral | Agree | Completely agree |
| 12. I can provide personalized home-based care services for older people whenever I use the Aifuxing App. | Completely disagree | Disagree | Neutral | Agree | Completely agree |
| Perceived ease of use | | | | | |
| 13. I am comfortable with my ability to use the Aifuxing App. | Completely disagree | Disagree | Neutral | Agree | Completely agree |
| 14. Learning to use the Aifuxing App is easy for me. | Completely disagree | Disagree | Neutral | Agree | Completely agree |
| 15. I can easily become skillful at using the Aifuxing App. | Completely disagree | Disagree | Neutral | Agree | Completely agree |
| 16. I find the Aifuxing App easy to use. | Completely disagree | Disagree | Neutral | Agree | Completely agree |
| 17. I can always remember how to log on to and use the Aifuxing App. | Completely disagree | Disagree | Neutral | Agree | Completely agree |
| User control | | | | | |
| 18. The Aifuxing App gives messages that tell me how to fix problems. | Completely disagree | Disagree | Neutral | Agree | Completely agree |
| 19. Whenever I make a mistake using the Aifuxing App, I recover easily and quickly. | Completely disagree | Disagree | Neutral | Agree | Completely agree |
| 20. The information (such as online help, on-screen messages, and other documentation) provided by the Aifuxing App is clear. | Completely disagree | Disagree | Neutral | Agree | Completely agree |

Health-ITUES-P, Health Information Technology Usability Evaluation Scale (Professional Care Provider Version).

Table S13. Path and standardized factor loadings of the Health-ITUES-R

| Path | | | Unstandardized estimate | Standardized estimate | S.E. | C.R. | p |
| --- | --- | --- | --- | --- | --- | --- | --- |
| AQ1 | <--- | Impact | 1.000 | 0.707 |  |  |  |
| AQ2 | <--- | Impact | 1.136 | 0.793 | 0.182 | 6.259 | *** |
| AQ3 | <--- | Impact | 1.009 | 0.710 | 0.167 | 6.048 | *** |
| AQ4 | <--- | Perceived Usefulness | 1.000 | 0.634 |  |  |  |
| AQ5 | <--- | Perceived Usefulness | 0.999 | 0.681 | 0.165 | 6.072 | *** |
| AQ6 | <--- | Perceived Usefulness | 0.904 | 0.644 | 0.156 | 5.807 | *** |
| AQ7 | <--- | Perceived Usefulness | 1.293 | 0.868 | 0.178 | 7.266 | *** |
| AQ8 | <--- | Perceived Usefulness | 1.080 | 0.729 | 0.169 | 6.405 | *** |
| AQ9 | <--- | Perceived Usefulness | 1.104 | 0.645 | 0.190 | 5.808 | *** |
| AQ10 | <--- | Perceived Usefulness | 1.121 | 0.678 | 0.185 | 6.048 | *** |
| AQ11 | <--- | Perceived Usefulness | 0.986 | 0.719 | 0.156 | 6.340 | *** |
| AQ12 | <--- | Perceived Usefulness | 0.705 | 0.586 | 0.131 | 5.366 | *** |
| AQ13 | <--- | Perceived Ease of Use | 1.000 | 0.779 |  |  |  |
| AQ14 | <--- | Perceived Ease of Use | 1.183 | 0.889 | 0.117 | 10.076 | *** |
| AQ15 | <--- | Perceived Ease of Use | 0.938 | 0.813 | 0.103 | 9.083 | *** |
| AQ16 | <--- | Perceived Ease of Use | 0.901 | 0.824 | 0.098 | 9.226 | *** |
| AQ17 | <--- | Perceived Ease of Use | 0.658 | 0.655 | 0.094 | 7.022 | *** |
| AQ18 | <--- | User Control | 1.000 | 0.813 |  |  |  |
| AQ19 | <--- | User Control | 0.816 | 0.692 | 0.130 | 6.264 | *** |
| AQ20 | <--- | User Control | 0.764 | 0.664 | 0.126 | 6.084 | *** |

The table summarizes the path and standardized factor loadings of the Health-ITUES-R in the confirmatory factor analysis; Health-ITUES-R, Health Information Technology Usability Evaluation Scale (Care Receiver Version); AQ, questions in the Care Receiver Version; ****p* < 0.001.

Table S14. Path and standardized factor loadings of the Health-ITUES-P

| Path | | | Unstandardized estimate | Standardized estimate | S.E. | C.R. | p |
| --- | --- | --- | --- | --- | --- | --- | --- |
| BQ1 | <--- | Impact | 1.000 | 0.785 | 0.126 | 8.869 | *** |
| BQ2 | <--- | Impact | 1.115 | 0.779 | 0.110 | 9.710 | *** |
| BQ3 | <--- | Impact | 1.073 | 0.851 |  |  |  |
| BQ4 | <--- | Perceived Usefulness | 1.000 | 0.851 | 0.083 | 12.157 | *** |
| BQ5 | <--- | Perceived Usefulness | 1.004 | 0.853 | 0.078 | 10.251 | *** |
| BQ6 | <--- | Perceived Usefulness | 0.801 | 0.768 | 0.083 | 10.287 | *** |
| BQ7 | <--- | Perceived Usefulness | 0.852 | 0.770 | 0.098 | 9.388 | *** |
| BQ8 | <--- | Perceived Usefulness | 0.917 | 0.725 | 0.096 | 8.927 | *** |
| BQ9 | <--- | Perceived Usefulness | 0.858 | 0.699 | 0.099 | 8.067 | *** |
| BQ10 | <--- | Perceived Usefulness | 0.798 | 0.650 | 0.076 | 10.056 | *** |
| BQ11 | <--- | Perceived Usefulness | 0.769 | 0.759 | 0.098 | 7.200 | *** |
| BQ12 | <--- | Perceived Usefulness | 0.708 | 0.595 |  |  |  |
| BQ13 | <--- | Perceived Ease of Use | 1.000 | 0.760 | 0.102 | 9.441 | *** |
| BQ14 | <--- | Perceived Ease of Use | 0.966 | 0.845 | 0.135 | 8.635 | *** |
| BQ15 | <--- | Perceived Ease of Use | 1.164 | 0.776 | 0.103 | 8.762 | *** |
| BQ16 | <--- | Perceived Ease of Use | 0.904 | 0.786 | 0.101 | 6.866 | *** |
| BQ17 | <--- | Perceived Ease of Use | 0.693 | 0.629 |  |  |  |
| BQ18 | <--- | User Control | 1.000 | 0.904 | 0.096 | 10.037 | *** |
| BQ19 | <--- | User Control | 0.966 | 0.780 | 0.085 | 8.979 | *** |
| BQ20 | <--- | User Control | 0.763 | 0.718 |  |  |  |

The table summarizes the path and standardized factor loadings of the Health-ITUES-P in the confirmatory factor analysis; Health-ITUES-P, Health Information Technology Usability Evaluation Scale (Professional Care Provider Version); App, application; BQ, questions in the Professional Care Provider Version; ****p* < 0.001.

Table S15. Patient Acceptance Questionnaire for Mobile Health Application

| Content | Extent | | | | |
| --- | --- | --- | --- | --- | --- |
| 1.Usefulness | | | | | |
| 1.1 This software can help me better understand diseases. | Strongly Disagree | Disagree | Neutral | Agree | Strongly Agree |
| 1.2 This software facilitates communication with nurses for me. | Strongly Disagree | Disagree | Neutral | Agree | Strongly Agree |
| 1.3 This software helps me adhere to medication schedules. | Strongly Disagree | Disagree | Neutral | Agree | Strongly Agree |
| 1.4 This software motivates me to engage in rehabilitation exercises. | Strongly Disagree | Disagree | Neutral | Agree | Strongly Agree |
| 1.5 This software enables me to resolve issues more quickly. | Strongly Disagree | Disagree | Neutral | Agree | Strongly Agree |
| 2.Ease of use | | | | | |
| 2.1 This software functions properly on my mobile phone. | Strongly Disagree | Disagree | Neutral | Agree | Strongly Agree |
| 2.2 The login process for this software is convenient. | Strongly Disagree | Disagree | Neutral | Agree | Strongly Agree |
| 2.3 It is easy to switch between different functions of this software. | Strongly Disagree | Disagree | Neutral | Agree | Strongly Agree |
| 2.4 I can easily find the information I need within this software. | Strongly Disagree | Disagree | Neutral | Agree | Strongly Agree |
| 2.5 The operational methods of this software are not complicated. | Strongly Disagree | Disagree | Neutral | Agree | Strongly Agree |
| 2.6 Without the guidance of a nurse, I would not be able to operate this software. | Strongly Disagree | Disagree | Neutral | Agree | Strongly Agree |
| 3.System/Interface | | | | | |
| 3.1 The interface layout of this software is clear. | Strongly Disagree | Disagree | Neutral | Agree | Strongly Agree |
| 3.2 The color scheme of this software interface is harmonious. | Strongly Disagree | Disagree | Neutral | Agree | Strongly Agree |
| 3.3 The font size in this software is suitable for my reading. | Strongly Disagree | Disagree | Neutral | Agree | Strongly Agree |
| 3.4 The relevant knowledge about diseases within this software is easily understandable. | Strongly Disagree | Disagree | Neutral | Agree | Strongly Agree |
| 3.5 When the internet is working properly, the operational speed of this software is acceptable. | Strongly Disagree | Disagree | Neutral | Agree | Strongly Agree |
| 3.6 This software rarely experiences abnormal situations during usage, such as frozen interfaces or automatic shutdowns. | Strongly Disagree | Disagree | Neutral | Agree | Strongly Agree |
| 3.7 It is not easy to make mistakes when entering values into the monitoring module, and there are warning alerts if abnormal values are entered, such as blood pressure exceeding normal ranges. | Strongly Disagree | Disagree | Neutral | Agree | Strongly Agree |
| 4.Reliability | | | | | |
| 4.1 I trust the nurses communicating with me through this software. | Strongly Disagree | Disagree | Neutral | Agree | Strongly Agree |
| 4.2 I believe in the disease-related knowledge presented within this software. | Strongly Disagree | Disagree | Neutral | Agree | Strongly Agree |
| 4.3 I do not worry about personal information security issues when using this software. | Strongly Disagree | Disagree | Neutral | Agree | Strongly Agree |
| 4.4 I think the app platform can properly safeguard the medical records data that I upload. | Strongly Disagree | Disagree | Neutral | Agree | Strongly Agree |
| 4.5 Through the use of this software, I have confidence in the recovery from my illness. | Strongly Disagree | Disagree | Neutral | Agree | Strongly Agree |
| 5.Usage attitude | | | | | |
| 5.1 I really like the features of this software. | Strongly Disagree | Disagree | Neutral | Agree | Strongly Agree |
| 5.2 I feel that this software meets my needs. | Strongly Disagree | Disagree | Neutral | Agree | Strongly Agree |
| 5.3 I am very satisfied with the user experience of this software. | Strongly Disagree | Disagree | Neutral | Agree | Strongly Agree |
| 5.4 I am highly pleased with the convenience provided by this software. | Strongly Disagree | Disagree | Neutral | Agree | Strongly Agree |
| 5.5 Through the services provided by this software, I feel cared for. | Strongly Disagree | Disagree | Neutral | Agree | Strongly Agree |
| 6.Usage tendency | | | | | |
| 6.1 I am willing to receive professional guidance from nurses through this software. | Strongly Disagree | Disagree | Neutral | Agree | Strongly Agree |
| 6.2 I believe that this service model is also valuable for other diseases. | Strongly Disagree | Disagree | Neutral | Agree | Strongly Agree |
| 6.3 I am willing to download and use this software. | Strongly Disagree | Disagree | Neutral | Agree | Strongly Agree |
| 6.4 I am willing to recommend this software to other elderly individuals. | Strongly Disagree | Disagree | Neutral | Agree | Strongly Agree |
